# Supplementary material for: Functional Innovation through Gene Duplication Followed by Frameshift Mutation
Source: Genes (Basel). 2022 Jan 21;13(2):190. doi: 10.3390/genes13020190 (PMC8872073; doi:10.3390/genes13020190)
Supplement: Supplementary file 1 [file genes-13-00190-s001.zip › Supplementary Figure S1.pdf]

genes

MDPI

Supplementary

Frequently Functional Innovation through Gene Duplication Followed by Frameshift Mutation

Baocheng Guo [1,2,3,*], Ming Zou [1], Takahiro Sakamoto [4] and Hideki Innan [4,*]

1 Key Laboratory of Zoological Systematics and Evolution, Institute of Zoology, Chinese Academy of Sciences, Beijing 100101, China; zoumingr@163.com
2 University of Chinese Academy of Sciences, Beijing 100049, China
3 Center for Excellence in Animal Evolution and Genetics, Chinese Academy of Sciences, Kunming 650223, China
4 Graduate University for Advanced Studies, Hayama, Kanagawa 240-0193, Japan; sakamoto_takahiro@soken.ac.jp
* Correspondence: guobaocheng@ioz.ac.cn (B.G.), innan_hideki@soken.ac.jp (H.I.)

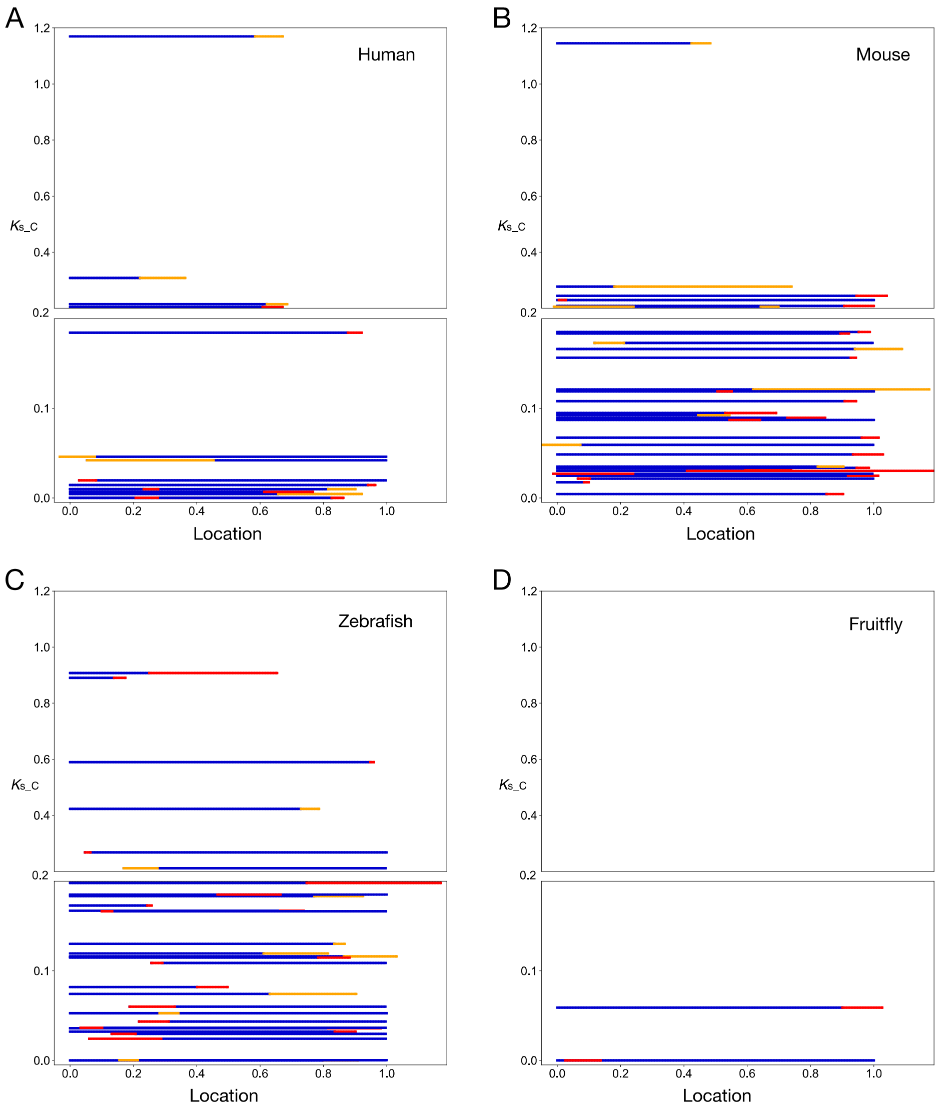

Figure S1. Relative location of the common frame region and frameshifted region in the derived copy, which are mapped on the (0,1) interval that corresponds to the entire coding region of the original copy, in human (**A**), mouse (**B**), zebrafish (**C**), and fruitfly (**D**). The vertical axis is *Ks_C*. The common frame region is presented in blue. If the ancestral/derived copies are specified by an outgroup gene, the frameshifted region is in red, otherwise in orange by assuming that the shorter gene is the derived copy (see text).
